# Sleep

**Published:** 1890-04

**Authors:** 


### SLEEP.

Up to the fifteenth year most young people require ten hours' sleep, and until the twentieth year nine hours. After that age every one finds out how much he or she requires, though as a general rule, at least six or eight hours are necessary. Eight hours sleep will prevent more nervous derangements in women than any medicine can cure. During growth there must be ample sleep if the brain is to develop to its full extent ; and the more nervous, excitable or precocious a child is, the more sleep should it get, if its intellectual progress is not to come to a premature standstill, or its life cut short at an early age.

